# Bitumen from the Dead Sea in Early Iron Age Nubia

**DOI:** 10.1038/s41598-020-64209-8

**Published:** 2020-05-20

**Authors:** Kate Fulcher, Rebecca Stacey, Neal Spencer

**Affiliations:** 1 Scientific Research, The British Museum, Great Russell St, London, WC1B 3DG UK; 2 Keeper of Nile Valley & Mediterranean Collections, The British Museum, London, WC1B 3DG UK

**Keywords:** Biogeochemistry, Materials science, Molecular biology

## Abstract

Bitumen has been identified for the first time in Egyptian occupied Nubia, from within the town of Amara West, occupied from around 1300 to 1050 BC. The bitumen can be sourced to the Dead Sea using biomarkers, evidencing a trade in this material from the eastern Mediterranean to Nubia in the New Kingdom or its immediate aftermath. Two different end uses for bitumen were determined at the site. Ground bitumen was identified in several paint palettes, and in one case can be shown to have been mixed with plant gum, which indicates the use of bitumen as a ground pigment. Bitumen was also identified as a component of a friable black solid excavated from a tomb, and a black substance applied to the surface of a painted and plastered coffin fragment. Both contained plant resin, indicating that this substance was probably applied as a ritual funerary liquid, a practice identified from this time period in Egypt. The use of this ritual, at a far remove from the royal Egyptian burial sites at Thebes, indicates the importance of this ritual as a component of the funeral, and the value attributed to the material components of the black liquid.

## Introduction

Black materials were excavated from different contexts in the pharaonic town of Amara West in Upper Nubia, dating from  around 1300 to 1050 BC  (19th–20th dynasties), and its cemeteries (1250–800 BC). The materials were of three types: black paints on ceramic sherds used as palettes; a black coating on a coffin plaster fragment; and a black friable material excavated from a tomb. Considering a group of contextually different black materials from a single site together offers an opportunity to understand the cultural and practical preferences for the choice of materials used to make black substances, and in which contexts they might be applied. Molecular analysis using gas chromatography-mass spectrometry (GC-MS) was used to identify the components of the different materials using three separate methods. Identifying the components allows the ingredients of the black substances to be connected to their end uses both in practical and symbolic terms.

### Amara West

**Figure 1:**
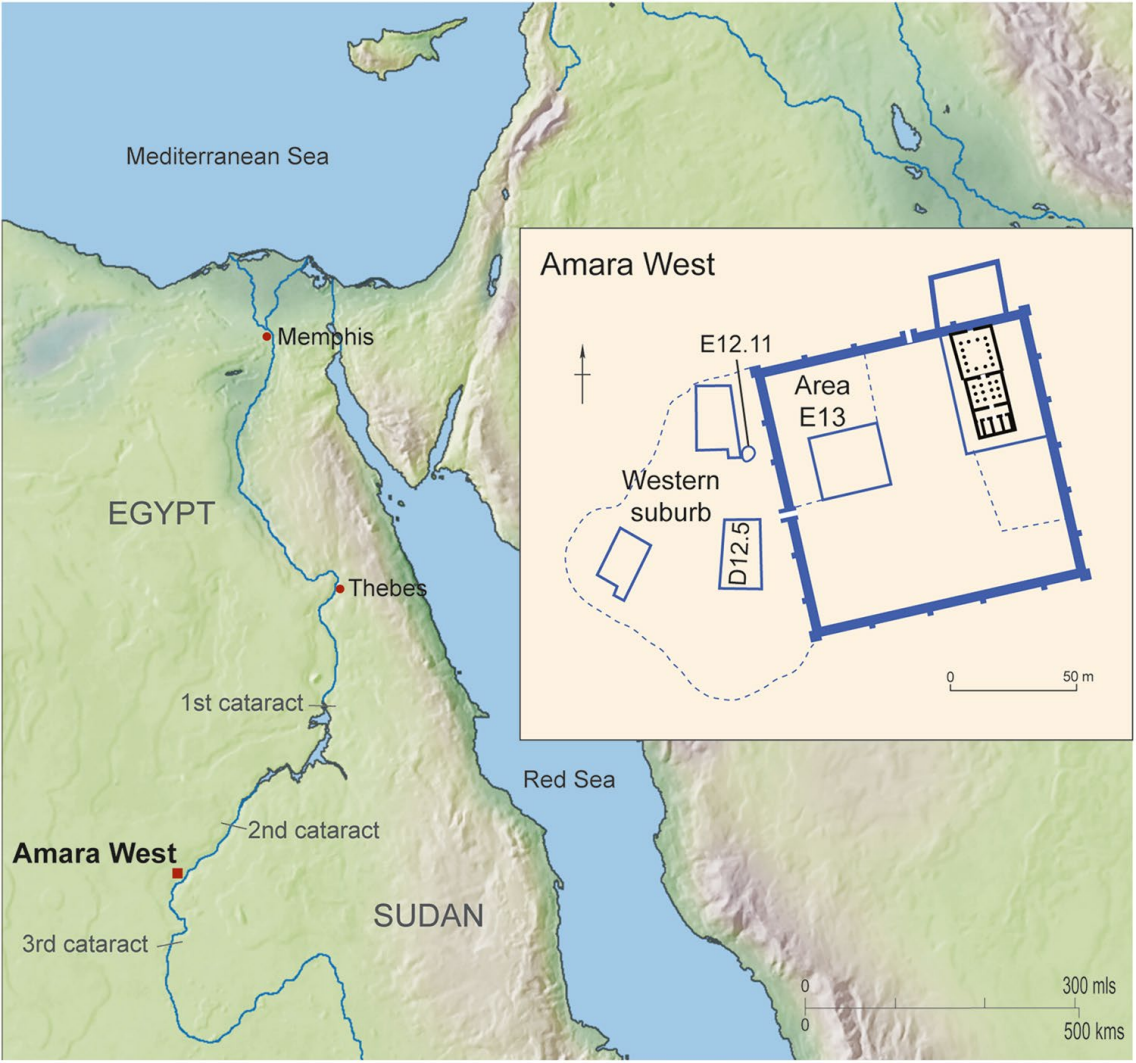
Map of Egypt and Sudan showing the location of Amara West, and [inset] layout of the site of Amara West. Image: Amara West Project, Trustees of the British Museum. The map was created using Adobe Illustrator 2019 adobe.com.

Amara West lies between the Second and Third Nile Cataracts, in the heart of Nubia, a region that stretched from Aswan in southern Egypt southwards to the Sixth Nile Cataract^1^ (Fig. 1). This region was intermittently occupied by pharaonic Egypt in the third and second millennium BC; during the New Kingdom (c. 1548–1086 BC), pharaonic towns were founded to control and administer resource extraction^2^. First excavated by the Egypt Exploration Society between 1939 and 1950^3^, fieldwork was then undertaken at Amara West by the British Museum from 2008 to 2019^4–6^. The town, founded around 1300BC (on the basis of inscriptional evidence), comprised a sandstone temple, a governor’s residence, storage facilities, and housing, set within a 108 m square enclosure wall^2^ (Fig. 1). From the late 19th Dynasty residents of the town began to build larger and more spacious houses outside the town wall, an area designated by the excavators as the “western suburb”^7,8^. While the latest architecture identified in the town dates to the late New Kingdom, ceramics scattered on the surface and burials in the associated cemeteries suggest a population living here, or nearby, through to the 8th century BC^9^.

### Painting materials

**Figure 2:**
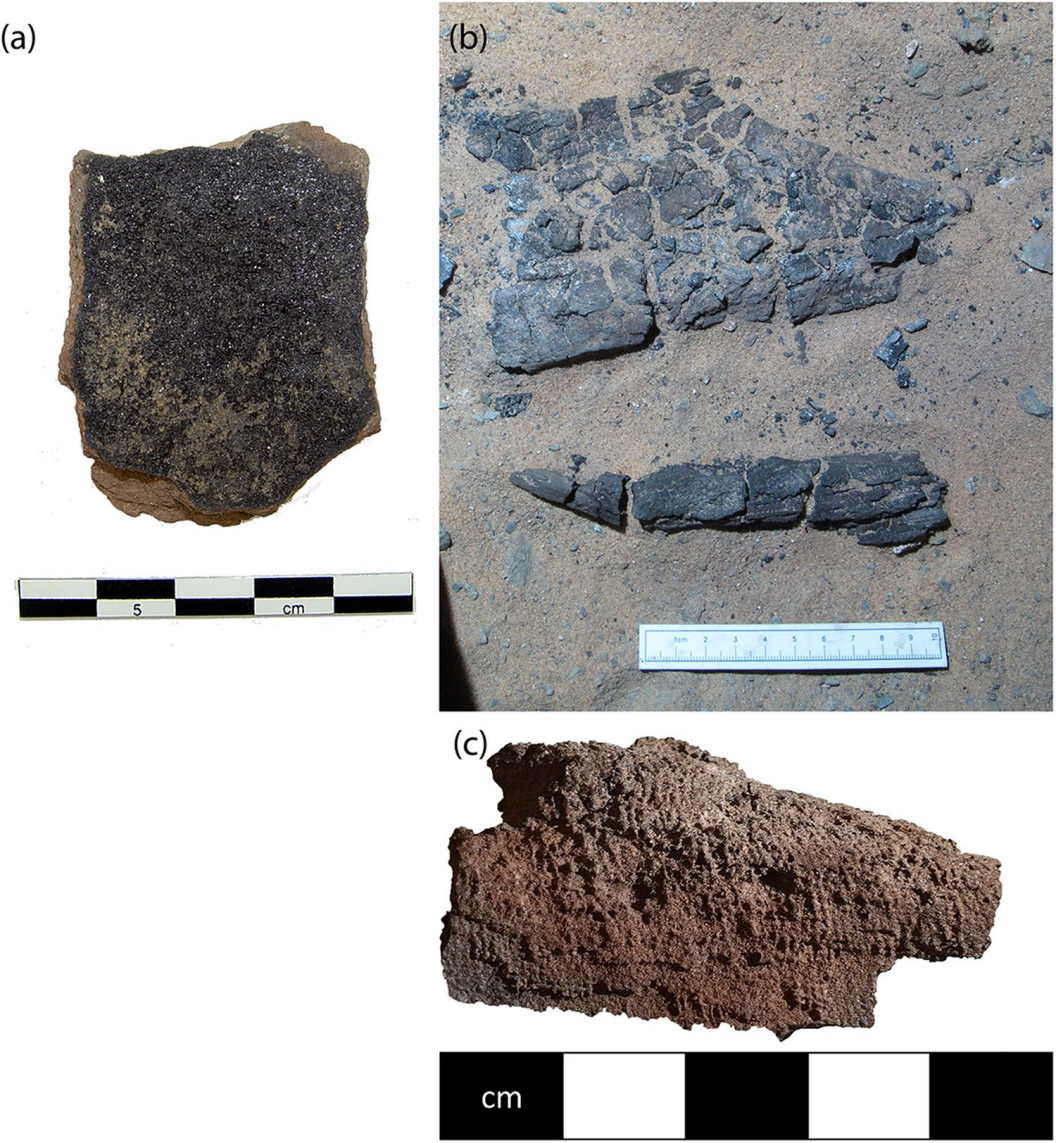
(**a**) Black paint in palette F6167, from magazine E13.14.2 in the walled town (**b**) Friable black solid F8623 as uncovered  in tomb G321  in cemetery D (**c**) Friable black solid from G321 with textile impressions. Photos: Amara West Project, Trustees of the British Museum.

Large quantities of painting materials were discovered in the form of ceramic sherds used as paint palettes (Fig. 2a), lumps of pigment, and grindstones, in an area at the front of storage magazines, which was possibly being used as a working area (E13.14)^10^. These magazines date to an early phase of the walled town in the 19th Dynasty, c. 1250 BC. Further finds, though in lower numbers, of palettes and pigments were found throughout the western suburb, which was constructed from the end of the 19th Dynasty and inhabited through the 20th Dynasty and later, c. 1180 BC to 1000BC. The colours of paint found in the palettes were red, yellow, white, black, blue, and green. Over 900 palettes were discovered in total, 31 containing black paint. No lumps of black pigment were identified, although charcoal was frequently found at the site.

### Funerary materials

Two cemeteries are associated with Amara West, referred to as Cemeteries C and D. A large rock-cut  tomb (G321) in Cemetery D, originally marked with a pyramid, was first used  for burials in the 20th Dynasty (c. 1190–1086 BC), with further use of the tomb into the 8th century BC. The archaeological deposits within, which have been disturbed in modern times (as evidenced by the presence of cigarette butts), included bone, wooden fragments and small pieces of painted plaster from coffins, and a large quantity of chunks of friable black material (F8623). The black material was scattered about in the fill, but one layer was preserved spread over an area approximately 20 cm square next to skeletal remains (Fig. 2b). The thickness was about 2 cm. The larger chunks retained textile impressions, indicating that it originally adhered to a textile, possibly wrappings on a body (Fig. 2c); the excavation records state that “bitumen with traces of wrapping” were found in this context. The chunks are too small to be able to tell if the wrappings adhered to the outside or inside of the textile. Either way, it appears that they formed part of the preparation of the body for burial, examples of this practice are known from other coffins (e.g. coffin EA20744 in the British Museum^11^).

A fragment of painted plaster from a coffin (F9743) found in tomb G244 in Cemetery C (19th–20th Dynasty) preserved a layer of a painted black substance, thicker than the usual decorative paint. The original scope of this black layer is not clear from the fragmentary state of the coffin remains, but it appears to have been applied to the exterior of the coffin over the outer plaster layer.

## Results

Five samples of black paint were taken from palettes from the early walled town, and five from palettes from the Western Suburb. Two samples of applied black material were taken from coffin fragments from tomb G244, and five further samples were taken from the black friable lumps found in tomb G321. One reference sample of archaeological Dead Sea bitumen from the British Museum Reference Collection was analysed alongside the samples. This material was procured from Tel Aviv University in the 1970s. Results were compared to data in the literature. The presence in each sample of bitumen, lipids, and gums, determined using separate methods, is shown in Table 1.

**Table 1:** Samples analysed by GC-MS. E and D designate grid numbers at Amara West. Check mark indicates compound class was identified; Tr = trace; n = none detected; blanks indicate that the sample was not tested.

Sample number	Find number	Archaeological context	Date	Analysis		
				Method A - Bitumen	Method B - Lipid	Method C - Gum
**Paint from palettes at Amara West**						
PS119		Magazine E13.14.1 [context 5230], walled town	c. 1200 BC	✓	n	n
PS121		Magazine E13.14.7 [context 5243], walled town	c. 1180 BC	✓	n	✓
PS139	F6281	Magazine E13.14.6 [context 5246], walled town	c. 1180 BC	n	n	n
PS415	F6167	Magazine E13.14.1 [context 5348], walled town	c. 1180 BC	✓	n	
PS152		Magazine E13.14.61 [context 5284], walled town	c. 1250 BC	✓	n	
PS861	F15279	House D11.2.5 [context 2772], western suburb	c. 1100 BC	Tr	n	
PS864	F15670	House D11.2.6 [context 2738], western suburb	c. 1000 BC	Tr	n	
PS873	F15666	House D12.12.3 [context 12346], western suburb	c. 1100–1000 BC	Tr	n	
PS877	F15137	House D12.7.6 [context 12062], western suburb	c. 1000 BC	Tr	n	
PS879	F15132	House D12.7.7 [context 12111], western suburb	c. 1100 BC	Tr	n	
**Black layer from coffin fragments**						
PS295	F9743	Tomb G244 [context 9515], Cemetery C	1250–1050 BC	✓	✓	
PS297	F9735	Tomb G244 [context 9511], Cemetery C	1250– 1050 BC	✓		
**Friable black material from tomb**						
AS1941		Tomb G321 [context 8455], Cemetery D	c. 1190–800 BC	✓	n	
AS1994		As above	c. 1190–800 BC	✓	✓	
AS1948		As above	c. 1190–800 BC	✓	Tr	
AS1949		As above	c. 1190–800 BC	✓	n	
AS1932		As above	c. 1190–800 BC	✓	✓	
AS1933		As above	c. 1190–800 BC	✓	✓	

### Results - Bitumen

**Figure 3:**
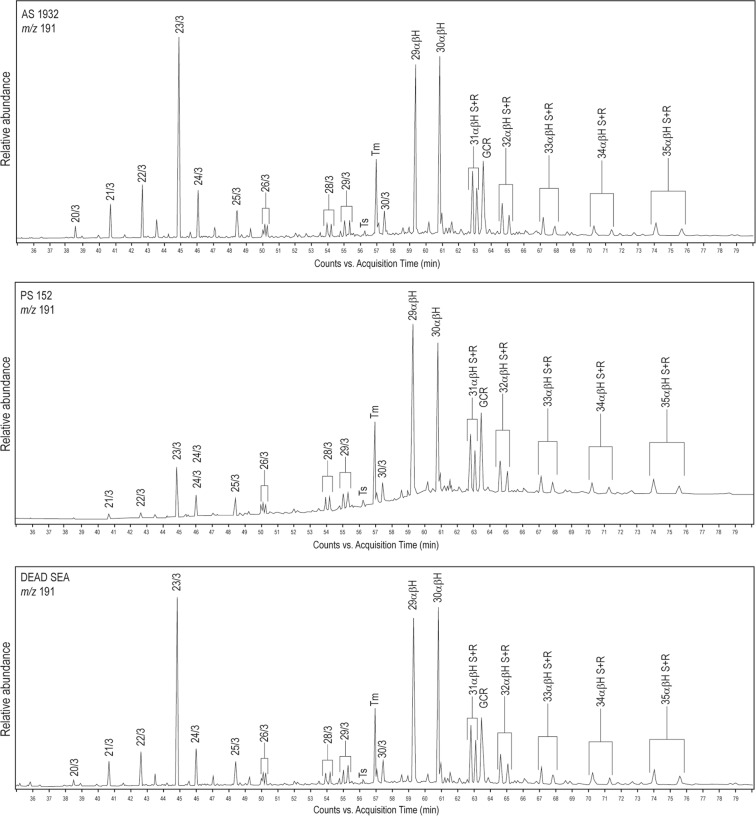
Mass chromatogram for ion m/z 191 (terpanes and hopanes) for AS1932 from G321 (friable solid), palette PS152, and reference sample from the Dead Sea, showing positions of terpanes (20/3 to 30/3), hopanes (29αβH to 34αβH; hopanes 31–34 are split into S and R), Ts, Tm, and gammacerane (GCR).

**Figure 4:**
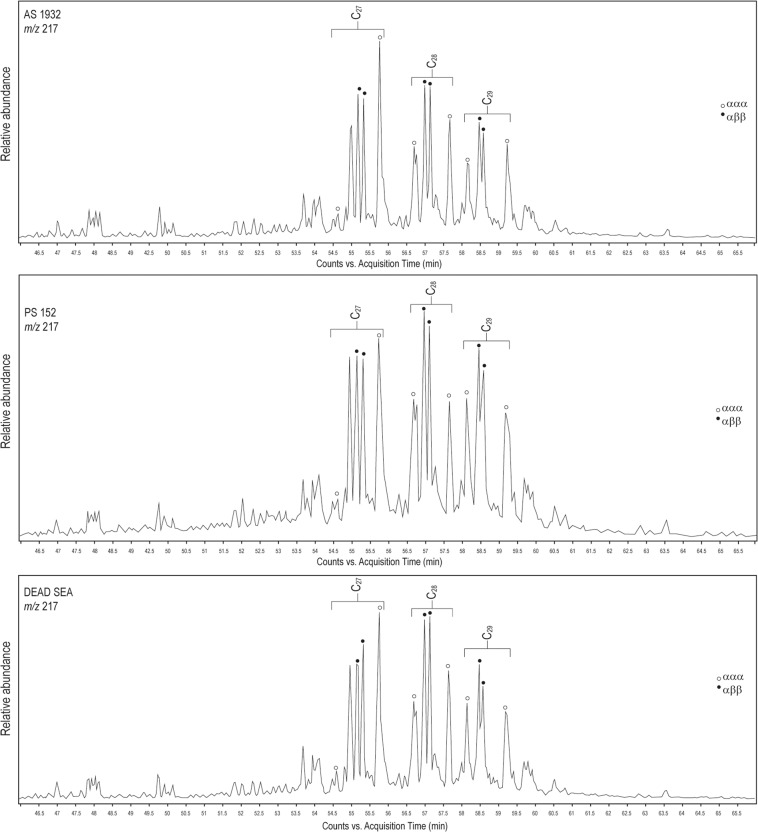
Mass chromatogram for ion m/z 217 for AS1932 from G321, palette PS152, and reference sample from the Dead Sea, showing positions of steranes.

Positive identification of bitumen in the samples analysed was determined by the presence of ions m/z 191 (hopanes) and m/z 217 (steranes)^12–14^, as shown in Figs. 3 and 4. Hopanes are abundant molecular fossils derived from terpenoids in bacteria and present in nearly all organic sediments^15^. Steranes are four-ring hydrocarbons derived from the degradation of steroids and sterols found in most higher plants and algae (but rare or absent from bacteria) via diagenesis and thermal maturation^16^.

Of the 10 samples of paint from palettes (PS numbers), 9 were found to contain bitumen, although those from the suburb only in trace amounts. All samples from the friable solid in tomb G321 (AS numbers) were found to contain bitumen. Samples PS295 and PS297 from the painted coffin fragments in G244 also contained bitumen.

**Figure 5:**
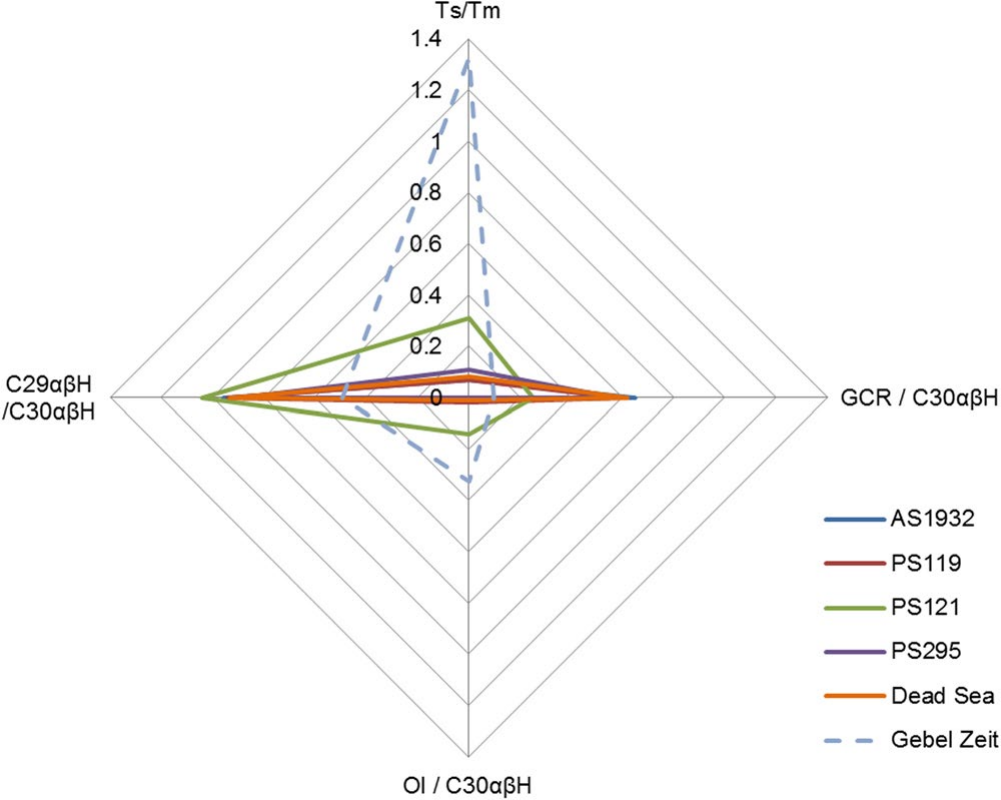
Radar plot for samples AS1932 from G321, palette PS119, black layer from coffin fragments PS295 (all group A), and palette PS121, as well as a reference sample from the Dead Sea, and data from Gebel Zeit^37^. See Table 2 for abbreviations.

Ratio data for a range of bitumen biomarkers are shown in Table 2. The results given in Table 2 are for the samples which provided good enough chromatograms from which to take data. Other samples showed hopanes and steranes but at trace levels such that peaks could not be reliably integrated. The ratios were calculated using the areas under the peaks obtained by manual integration. Selected ratios are plotted in Fig. 5 with comparative reference data.

**Table 2:** Results of GC-MS analysis of Amara West and reference samples, biomarkers. Pr/Ph = pristane/phytane, data taken from total ion chromatogram (TIC). All other data from m/z 191: Ts = 18α(H)-22,29,30-trinorhopane, Tm = 17α(H)-22,29,30-trinorhopane; GCR = gammacerane; Ol = oleanane; CnαβH = 17α,21β-hopane at Cn; C31R = 17α,21β-22R-30-homohopane at C31; C29αβH = 17α,21β-norhopane at C29; Cn = 17α,21β-22S + R-29-homohopane at Cn; C26/C25 TT = C26/C25 tricyclic terpanes. ND = no data.

	Pr/Ph (TIC)	Ts/Tm (m/z 191)	GCR/C30αβH (m/z 191)	Ol/C30αβH (m/z 191)	C31R/C30αβH (m/z 191)	C29αβH /C30αβH (m/z 191)	C35/C34 (m/z 191)	C26/C25 TT (m/z 191)
AS1932	0.22	0.07	0.65	0.00	0.31	0.96	1.30	0.24
AS1933	0.24	0.07	0.67	0.00	0.32	0.90	1.48	0.35
AS1948	0.19	0.11	0.65	0.03	0.34	0.86	1.45	0.40
AS1949	0.19	0.08	0.73	0.03	0.38	0.98	1.47	0.36
AS1994	0.19	0.07	0.68	0.01	0.33	0.88	1.41	0.32
PS295	ND	0.11	0.58	0.00	0.28	0.90	1.23	0.41
PS152	ND	0.05	0.65	0.00	0.31	0.88	1.88	0.81
PS121	0.07	0.31	0.25	0.14	0.53	1.04	nd	0.34
PS119	ND	0.07	0.59	0.02	0.34	0.94	1.85	0.16
PS415	ND	0.09	0.92	0.04	0.31	1.68	1.98	0.37
Dead Sea reference	0.20	0.08	0.62	0.01	0.32	0.93	1.52	0.49
Gebel Zeit^37^	1.3	1.9	0.11	0.26	ND	0.4	0.8	ND

### Results - Lipids

**Figure 6:**
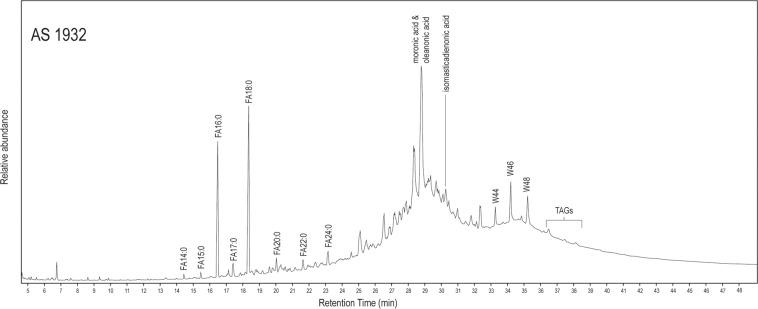
Chromatogram for AS1932 (Method B1). FAn:0 = saturated fatty acids with n number of carbons; Wn = wax ester with n number of carbons.

The paint samples from the palettes (PS numbers) contained no resins, oils, fats, or waxes. Analysis results from G321 material (AS numbers) varied, due to the fact that this substance was not homogenous (Supplementary Table S1). Slightly different results were obtained for AS1932 and AS1994 by the two GC methods, most likely due to sample heterogeneity. AS1932 (Fig. 6), AS1933, and AS1994 contained several organic products. The presence of fatty acids, with stearic acid predominating, traces of triacylglycerols, and no diacids, suggests an animal fat^17,18^. Wax esters with carbon chain length 42, 44, 46, and 48, and long chain fatty acids, evidence a natural wax component^19,20^. The mass spectra of the wax esters have a peak at m/z 257 indicating an acid moiety with 16 carbons, thus are a series of even carbon number long-chain palmitate wax esters, probably indicating the presence of beeswax^21^, although no alcohols or alkanes were detected, possibly due to heating in ancient times^19^. In the mass spectra of the wax esters of carbon chain length 44, 46 and 48, m/z 257 is the base peak and there is also a peak at m/z 285 (Supplementary Fig. S1). For the ester with 42 carbons observed in the chromatogram for AS1933 m/z 257 is present but the base peak is m/z 285. The m/z 285 ion indicates an acid moiety in the wax ester with 18 carbons, suggesting the presence of another waxy material, possibly plant based^22^. Peaks for oleanonic acid and moronic acid, with traces of masticadienonic and isomasticadienonic acids indicate that the resin from *Pistacia* sp. was a component of the mixture^23–25^. The samples taken from AS1941, AS1948, and AS1949 gave very poor chromatograms for lipids. Sample PS295 from the coffin gave a poor chromatogram with a peak for moronic acid, indicating that this material also contained pistacia resin. Given the heterogeneity of the samples from G321, it is possible that a larger sample from this coffin may have included a wider range of ingredients.

### Results - Gums

Plant gums are sugary substances composed of monosaccharides. Identifying the range of monosaccharides present can sometimes enable the identification of the plant from which the gum was taken; published analyses of plant gums report the presence of the monosaccharides arabinose, fucose, xylose, mannose, rhamnose, galactose and glucose in varying quantities^26–29^.

Of the three samples from palettes analysed for monosaccharides, one (PS121) contained fucose, mannose, galactose, and other unidentified sugars (Supplementary Fig. S2), which indicates that plant gum was used as a binder with the black pigment. The other two were either not mixed with a gum or the material was too degraded to be detected using this method. The presence of fucose suggests that the gum is tragacanth, obtained from the roots of *Astragalus* sp., but the presence of mannose points to a fruit gum, although mannose has been reported in tragacanth gum by one study^26,29,30^. *Astragalus* sp. grows in Turkey, Syria, Iraq and Iran, and would have been imported into Egypt^31^. A similar result was reported for paint samples from one New Kingdom object (17, mummy mask) and two Third Intermediate Period objects (21, mummy mask; 22, falcon) from the Museum of Fine Arts, Boston^32^. The authors concluded that the binders may have included tragacanth but were probably a mixture of gums^32^.

## Discussion

### Source of bitumen

Bitumen is composed of geologically old organic matter; its molecular make-up depends on the original living organisms that decayed to create it, which varies between formations^33^. Biomarkers are the “molecular fossils” from these organisms that are present in petroleum products, and can be used to identify types of source rock of petrochemicals and to match compounds from the same source^14^. A range of biomarkers should be considered because it is sometimes unclear to what extent each biomarker can predict the depositional environment^14,34^, and there are further issues with archaeological samples such as alterations to the chemical structure of the samples due to their archaeological depositional environment (rather than the geological one which formed the bitumen), and contamination from other substances. In addition, the pattern of hopanes (m/z 191) and steranes (m/z 217) can be studied for similarities with samples of known origin^35,36^.

Each biomarker is difficult to interpret independently, but the cumulative evidence seems to point to a marine carbonate source rock for most of the Amara West bitumen (Supplementary Table S2). The chromatograms of the friable material from G321, PS295 from the coffin, and palettes PS119, PS152, and PS415 (herein referred to as Group A) show a very similar pattern. Gammacerane (GCR) is prominent for Group A, and oleanane absent or very low. Diasteranes (m/z 259) are very low or absent for all of Group A. The patterns of the chromatograms for Group A are similar to those obtained for the Dead Sea from the reference sample and in the literature (Figs. 3 & 4). Dead Sea bitumen is characterised by a low pristane/phytane ratio of around 0.5, very low or absent diasteranes, low oleanane, high gammacerane, high C35/C34 homohopanes, and a complete series of tricyclic terpanes (C19–C30), maximizing at C23^35,37–39^. Ts/Tm varies slightly for Dead Sea samples reported in the literature, from 0.04 in geological samples to 0.08 in archaeological samples^13,35^. Most of the samples analysed from Amara West fit into this range, with the exception of PS121.

The black pigment from Amara West palette PS121 has different biomarker values from the other Amara West samples (Table 2). The oleanane index is higher (0.14 compared to average 0.013 from the AS samples from G321), and the gammacerane index lower (0.25 compared to average 0.67 from the AS samples from G321; Table 2). These results are unlikely to be explained by biodegradation, as it would be expected that both the oleanane and the gammacerane levels would be elevated^40^. It appears likely that this sample had a non-Dead Sea origin, especially considering the presence of oleanane, which is very low or absent for the Dead Sea and other Amara West samples. Another source of bitumen in Egypt is Gebel Zeit^37,41^, but as can be seen from Table 2 and the radar plot in Fig. 5, the data from Gebel Zeit does not match any of the samples in this study. The origin of the bitumen in PS121 cannot currently be identified, and may be from a mixture of sources, which would make definitive identification difficult.

### Significance of bitumen as a pigment

The ground black material from the palettes contained bitumen in 9 out of 10 cases, and one of these was shown to have been mixed with a plant gum binder to make a paint. This is the first molecular identification of bitumen being used as a ground pigment in a pharaonic context. There is one other published example of bitumen used as a pigment in Egypt prior to the Roman Period, on a 19th Dynasty model boat from Gurob, but this finding has not been confirmed by molecular analysis^42^. Most black paints and inks from ancient Egypt have been identified as carbon, obtained from burning organic matter^43–45^, but given the difficulties of distinguishing elemental carbon from bitumen, which is only really possible using molecular analysis (GC-MS), it is possible that the use of bitumen as a pigment in ancient Egypt has been under identified. The use of bitumen could have been a lot more extensive than is indicated by the evidence to date, and this should be taken into account in future pigment studies. While some of the samples come from occupation and rubbish deposits found within storage magazines (E13.14), and thus might reflect the  period when apparatus of the pharaonic state was most prevalent at Amara West (inscriptions, storage facilities, seal impressions), other instances were identified in the latest phases of occupation, suggesting a continuing  use of bitumen as a pigment. Nonetheless, the Dead Sea origin of the bitumen indicates that this trade would have been coming from the north, through Egypt.

Given the ease with which carbon can be obtained, i.e. by burning anything organic, the use of bitumen as a pigment must have had a significance. We do not know the end-use of the black pigment in the palettes, but it is possible it was being reserved for a particular use.

### Significance of bitumen in a funerary context

The friable material from tomb G321 was found to consist of resin from *Pistacia* sp., a lipid component (fat or oil), wax, and bitumen. The composition of the G321 material is very similar to mummification “balms”, however the black material from Amara West was not found on the body or in body cavities, but instead in scattered fragments and in one area quite a thick, flat puddle close to but not on a (disturbed) body. The black material was applied as a liquid, which allowed textile impressions to form on the surface of the substance when it solidified. This suggests it may have been applied to the exterior of a wrapped body. Black ritually applied liquids are known from the exterior of wrapped bodies and funerary containers from Egypt, and are the subject of current research at the British Museum. Analyses of these externally applied black liquids has shown them to consist of lipids, beeswax, bitumen, and conifer resin or pistacia resin in various combinations (for example, EA6662, EA6660, EA48001, and EA24906 in the collections of the British Museum^46,47^). The components of the black material from Amara West and the context in which it was found are consistent with the Egyptian black funerary liquids. The black material from the coffin fragments from G244 also contained bitumen and pistacia resin, and appears to have been applied to the surface of the coffin. Given the similarity in components and context to the material in G321, it seems likely that this was also a ritually applied black liquid. The use of similar ingredients in mummification balms and black varnishes on funerary statues suggests that this black liquid had multiple uses in funerary practice^13,33,48–54^. A link to Osiris may be inferred from the colour of the substance, Osiris is known as “the black one” and shown with black skin, and from the similarity of the liquid to mummification balms, the deceased is identified as an aspect of Osiris^55^.

### Significance of bitumen at Amara West

The biomarkers in the black materials from Amara West are consistent with those of Dead Sea examples, which is likely to be evidence for a trade in solid bitumen from the Dead Sea into Nubia over the 19th and 20th dynasties (c. 1300 to 1070 BC). Evidence for the trade in bitumen into the Nile Valley during the New Kingdom has so far been very limited, so this would be a major contribution to this dataset. Alternatively, the bitumen found in G321 may relate to the later use of the tomb, in the period after Egyptian occupation, as ceramics diagnostic of that date and distinctive Nubian wooden funerary bed fragments were found in the same context. If this is the case, it may reflect the adoption, and perhaps reinterpretation, of Egyptian funerary practises by individuals who identified as Nubian. Previous studies have found bitumen in mummification materials from the Third Intermediate Period to the Roman Period (c. 1086 BC to 300 AD), most of which was shown to have come from the Dead Sea^13,33,41,56–60^, and a trade route for Dead Sea bitumen into Egypt in the 4th to 3rd millennium BC has been identified from lumps of archaeological bitumen^38^. Molecular evidence for bitumen from the New Kingdom (pre-dating the Third Intermediate Period) is limited to the black coating on the coffin of Henutmehyt in the British Museum (EA48001)^46^, the balm of a mummified man from Thebes^13^, an identification of Dead Sea bitumen in a 19th Dynasty “mummy balm”^12^, and the presence of hopanes in the black coatings on an 18th Dynasty canopic chest and anthropoid coffin^49^. Amara West was founded by the pharaonic state but the presence at the town of  individuals who identified as Nubian is suggested by the production and use of Nubian pottery, and building. E12.11 that reflects Nubian architectural traditions^9,61–63^. The evidence from the tombs at Amara West appears to demonstrate an integration of Egyptian funerary and technological traditions with those from Nubia, such as the tumulus superstructure over tomb G244, from which the coffin fragment comes^9^. In this context it is interesting to see evidence for the use of a ritual black liquid that is linked to the Egyptian embalming tradition.

## Conclusion

This study provides evidence of the use of bitumen from the Dead Sea and another origin, in Nubia during the early Iron Age pharaonic occupation of the region and its immediate aftermath. This bitumen was found in two contexts, ground and mixed with gum to make a black paint, and as a component of an organic ritual liquid.

The results provide the first molecular identification of bitumen used as a ground pigment in a pharaonic context, albeit outside Egypt itself. It is possible that bitumen has hitherto been under recognised as a painting material in the ancient Nile Valley and should be considered by future pigment studies.

The black substance from G321 and the coffin fragments are examples of an Egyptian funerary ritual using long-distance imported ingredients, for at least two individuals on two separate occasions. The use of this ritual at a far remove from the royal Egyptian burial sites at Thebes, and in graves reflecting the entanglement of Egyptian and Nubian funerary traditions, indicates the importance of this ritual as a component of the funeral, and the value attributed to the material components of the black liquid. The liquid probably had important ritual associations with Osiris, who is associated with the dead and the colour black.

Given that evidence for bitumen use in Egypt in the New Kingdom has previously been limited to a few individual samples from objects with poor provenance^12,13,42,46,49^, this study provides proof for a much more extensive use than might have been suspected, with a secure archaeological context.

## Methods

Three methods were used, each designed to analyse the samples for a different set of components: bitumen, lipids, and gums. The aim was to determine the extent of the use of bitumen at the site and whether the other components of the materials would provide clues for different end uses. Previous analysis of Egyptian paints has shown that plant gums were used as a paint binder^31,64–68^, whereas black funerary liquids were complex mixtures of organic products^13,46,50,52,53,56,69,70^. As stated in other publications^13,71^ the analysis method will determine the components that can be detected, so different methods were applied.

### Method A - bitumen analysis

Samples were dissolved in 1 ml dichloromethane (DCM), and heated at 40 °C for 2 hours, after which the solution was decanted, and dried under a stream of nitrogen. This was done 3 times, combining the extracts. 20 µl DCM and 1 ml hexane were added to the soluble fraction, the asphaltene fraction precipitated out, and this was left overnight to settle. The solution was then decanted and dried under a stream of nitrogen to obtain the maltene fraction.

Each maltene fraction was then fractionated using column chromatography. 100 µl hexane was added to the maltene fraction. Each was decanted into a glass pipette held upright and plugged with glass wool and half filled with dried silica (chromatography grade 60–120 µm, pre-extracted with DCM/methanol 97:3 (v:v), followed by hexane, then oven dried) to which hexane had been added to exclude moisture. The first fraction was extracted using 3 ml hexane washed through the pipette; the second using 3 ml DCM:hexane 1:3 (v:v); the third using 3 ml DCM:methanol 2:1 (v:v). The elutes were collected and dried in a stream of nitrogen. For analysis, 50 µl of hexane was added to the first fraction and this was decanted to a micro vial.

The GC-MS analysis was carried out with an Agilent HP5-MS column (30 m × 0.25 mm, 0.25 µm film thickness) with splitless injection, coupled to an Agilent 5973 MSD. The mass spectrometer was operating in the electron impact (EI) mode at 70 eV and scanning m/z 50 to 550 amu. The oven was set at 60 °C to 290 °C at 4 °C/min with the final temperature held for 30.5 mins. GC-MS analysis was run in two modes: scan and Selective Ion Monitoring (SIM). Acquisition in SIM mode targeted ions: 177, 191, 217, 218, 259.

### Method B - Lipid analysis

Samples were solvent extracted 3 times using DCM, as in Method A, and the resulting combined extracts were dried under a stream of nitrogen. Each was derivatised using 100 µl silylating reagent N,O-bis(trimethylsilyl)trifluoroacetamide (BSTFA) plus 1% trimethylchlorosilane (TMCS), heated at 70 °C for 1 hour. After cooling, samples in BSTFA were auto injected into the GC.

#### Method B1

The GC-MS analysis was carried out with a SGE HT5 GC column (12 m × 0.22 mm; 1 µm film thickness) with splitless injection, coupled to an Agilent 5975 C MSD. The mass spectrometer was operating in the electron impact (EI) mode at 70 eV and scanning m/z 50 to 1000. The oven was set at 50 °C to 370 °C at 10 °C per minute, isothermal for 15 minutes.

#### Method B2

Two further samples were taken from AS1932 and AS1994, which were prepared in the same way, and run on a longer method to further separate the peaks. The oven was set at 50 °C to 320 °C at 5 °C per minute and then 10 °C to 370 °C, isothermal for 15 minutes. PS295 was only run on the longer method, because the sample was taken at a later date.

Data were collected in scan and SIM mode. GC-MS analysis was run in two modes: scan and Selective Ion Monitoring (SIM). Acquisition in SIM mode targeted ions: 0–15 mins (aromatics) m/z 105, 205, 267, 297; 15–25 mins (conifer) m/z 219, 239, 253, 459; 25–35 mins (pistacia) m/z 189, 409, 421, 526.

### Method C - Gum analysis

#### Samples for gum analysis were taken from PS119, PS121 and PS139

The method followed for the analysis of Amara West paints for plant gums was the standard operating procedure used at the British Museum for the preparation of polysaccharide samples for GC-MS analysis of neutral sugars and uronic acids, based on a published method^26^.

Samples and reference samples were hydrolysed by the addition of 500 µl of 0.5 M hydrochloric acid and heated at 80 °C for 20 hours. The solution was decanted and dried under nitrogen. Samples were derivatised by the addition of 300 µl Sigma-Sil A (1:3:9 ratio of trimethylchlorosilane (TMCS), hexamethyldisilazane (HMDS) and pyridine), and heated at 80 °C for 2 hours. Samples were dried under nitrogen and dissolved in 100 µl hexane in preparation for injection into the GC-MS instrument. A blank and three reference samples were prepared alongside the samples using the same method.

The instrument and column used were the same as for the bitumen analysis. The oven was set at 40 °C to 130 °C at 9 °C/min, then to 290 °C at 2 °C/min, with the final temperature held for 10 mins.

In all cases the data were analysed using Masshunter software and the NIST database.

## Supplementary information

Supplementary Information.

## Data Availability

All relevant data are within the paper and its Supporting Information files. Archive documents referred to in the paper are held in the archives of the British Museum, Department of Scientific Research (Project Record no. 7671) and can be viewed in hard copy or electronically by appointment through science@thebritishmuseum.ac.uk.

## References

[1] Edwards, D. N. *The Nubian Past*. *An Archaeology of the Sudan* (Routledge, 2004).

[2] Spencer, N. Building on new ground: the foundation of a colonial town at Amara West, in *Nubia in the New Kingdom: Lived Experience, Pharaonic Control and Indigenous Traditions* (eds. Spencer, N., Stevens, A. & Binder, M.) 323–356 (Peeters, 2017).

[3] Spencer, P. *Amara West I: The Architectural Report* (Egypt Exploration Society, 1997).

[4] Spencer, N. Amara West: considerations on urban life in colonial Kush. In *The Fourth Cataract and beyond**. Proceedings of the 12th International Conference for Nubian Studies* (eds. Anderson, J. R. & Welsby, D. A.) 457–486 (Peeters, 2014).

[5] Spencer, N., Stevens, A. & Binder, M. *Amara West: Living in Egyptian Nubia* (British Museum, 2014).

[6] www.britishmuseum.org/AmaraWest (2020).

[7] Spencer, N. Cemeteries and a late Ramesside suburb at Amara West. *Sudan & Nubia* **13**, 47–61 (2009).

[8] Spencer, N. Creating a neighborhood within a changing town: household and other agencies at Amara West in Nubia. In *Household**Studies in Complex Societies: (Micro) Archaeological and Textual Approaches* (ed. Müller, M.) 169–210 (Oriental Institute of the University of Chicago, 2015).

[9] Binder, M. The New Kingdom tombs at Amara West: funerary perspectives on Nubian – Egyptian interactions. In* Nubia in the New Kingdom: Lived Experience, Pharaonic Control and Indigenous Traditions* (eds. Spencer, N., Stevens, A. & Binder, M.) 591–613 (Peeters, 2017).

[10] Fulcher, K. *Painting Amara West: the technology and experience of colour in New Kingdom Nubia*. PhD Thesis, UCL Institute of Archaeology (2018).

[11] Connan, J. *Le bitume dans l’Antiquité (Bitumen in antiquity)*. (Errance, 2012).

[12] Connan J, Dessort D (1991). Du bitume dans les baumes de momies égyptiennes (1295 av. J.-C.-300 ap. J.-C.): détermination de son origine et évaluation de sa quantité (Bitumen in the balms of Egyptian mummies (1295 BC-300 AD): determination of its origin and evaluation of its quantity). Comptes Rendus l’Académie des Sci. Série 2, mécanique. Phys. Chim. Sci. l’univers, Sci. la terre.

[13] Łucejko J, Connan J, Orsini S, Ribechini E, Modugno F (2017). Chemical analyses of Egyptian mummification balms and organic residues from storage jars dated from the Old Kingdom to the Copto-Byzantine period. J. Archaeol. Sci..

[14] Peters, K. E., Walters, C. C. & Moldowan, J. M. *The Biomarker Guide* (Cambridge University Press, 2005).

[15] Ourisson G, Albrecht P (1992). Hopanoids. 1. Geohopanoids: the most abundant natural products on Earth?. Acc. Chem. Res..

[16] Mackenzie A, Brassell S, Eglinton G, Maxwell J (1982). Chemical fossils: the geological fate of steroids. Science.

[17] Colombini, M. P., Modugno, F. & Ribechini, E. GC/MS in the characterization of lipids. In *Organic Mass Spectrometry in Art and Archaeology* (eds. Colombini, M. P. & Modugno, F.) 191–213 (Wiley, 2000).

[18] Evershed RP (2002). Chemistry of archaeological animal fats. Acc. Chem. Res..

[19] Heron C, Nemcek N, Bonfield KM, Dixon D, Ottaway BS (1994). The chemistry of neolithic beeswax. Naturwissenschaften.

[20] Regert M, Colinart S, Degrand L, Decavallas O (2001). Chemical alteration and use of beeswax through time: accelerated ageing tests and analysis of archaeological samples from various environmental contexts. Archaeometry.

[21] Evershed, R. P. Biomolecular analysis by organic mass spectrometry in Modern analytical methods in art and archaeology (eds. Ciliberto, E. & Spoto, G.) 177–240 (Wiley, 2000).

[22] Ribechini E, Modugno F, Colombini MP, Evershed RP (2008). Gas chromatographic and mass spectrometric investigations of organic residues from Roman glass unguentaria. J. Chromatogr. A.

[23] Modugno F, Ribechini E, Colombini MP (2006). Chemical study of triterpenoid resinous materials in archaeological findings by means of direct exposure electron ionisation mass spectrometry and gas chromatography/mass spectrometry. Rapid Commun. Mass Spectrom..

[24] Stern B, Heron C, Corr L, Serpico M, Bourriau J (2003). Compositional variations in aged and heated pistacia resin found in late bronze age canaanite amphorae and bowls from Amarna, Egypt. Archaeometry.

[25] Nicholson TM (2011). Enlightening the past: Analytical proof for the use of Pistacia exudates in ancient Egyptian embalming resins. J. Sep. Sci..

[26] Bleton J, Mejanelle P, Sansoulet J, Goursaud S, Tchapla A (1996). Characterization of neutral sugars and uronic acids after methanolysis and trimethylsilylation for recognition of plant gums. J. Chromatogr. A.

[27] Marinach C, Papillon M-C, Pepe C (2004). Identification of binding media in works of art by gas chromatography–mass spectrometry. J. Cult. Herit..

[28] Scott, D. Greener shades of pale: a review of advances in the characterisation of ancient Egyptian green pigments. In *Decorated Surfaces on Ancient Egyptian Objects: Technology**, Deterioration and Conservation*. Proceedings of a Conference Held in Cambridge, UK on 7-8 September 2007 (eds. Dawson, J., Rozeik, C. & Wright, M. M.) 32–45 (Archetype in association with the Fitzwilliam Museum and Icon Archaeology Group, 2010).

[29] Vallance SL, Singer BW, Hitchen SM, Townsend JH (1998). The development and initial application of a gas chromatographic method for the characterization of gum media. J. Am. Inst. Conserv..

[30] Al-Hazmi MI, Stauffer KR (1986). Gas chromatographic determination of hydrolyzed sugars in commercial gums. J. Food Sci..

[31] Newman, R. & Serpico, M. *Adhesives and binders in Ancient Egyptian Materials and Technology* (eds. Nicholson, P. T. & Shaw, I.) 475–494 (Cambridge University Press, 2000).

[32] Newman, R. & Halpine, S. M. *The binding media of ancient Egyptian painting in Colour and Painting in Ancient**Egypt* (ed. Davies, W. V.) 22–32 (British Museum Press, 2001).

[33] Connan J (1999). Use and trade of bitumen in antiquity and prehistory: molecular archaeology reveals secrets of past civilizations. Philos. Trans. R. Soc. B Biol. Sci..

[34] Mello MR, Gaglianone PC, Brassell SC, Maxwell JR (1988). Geochemical and biological marker assessment of depositional environments using Brazilian offshore oils. Mar. Pet. Geol..

[35] Connan J, Nissenbaum A (2004). The organic geochemistry of the Hasbeya asphalt (Lebanon): comparison with asphalts from the Dead Sea area and Iraq. Org. Geochem..

[36] Enkhtsetseg E, Byambagar B, Monkhoobor D, Avid B, Tuvshinjargal A (2011). Determination of sterane and triterpane in the Tamsagbulag oilfield. Adv. Chem. Eng. Sci..

[37] Barakat AO, Mostafa A, Qian Y, Kim M, Kennicutt MC (2005). Organic geochemistry indicates Gebel El Zeit, Gulf of Suez, is a source of bitumen used in some Egyptian mummies. Geoarchaeology.

[38] Connan J, Nissenbaum A, Dessort D (1992). Molecular archaeology: export of Dead Sea asphalt to Canaan and Egypt in the Chalcolithic-Early Bronze Age (4th-3rd millennium BC). Geochim. Cosmochim. Acta.

[39] Rullkötter J, Spiro B, Nissenbaum A (1985). Biological marker characteristics of oils and asphalts from carbonate source rocks in a rapidly subsiding graben, Dead Sea, Israel. Geochim. Cosmochim. Acta.

[40] Blanc P, Connan J (1992). Origin and occurrence of 25-norhopanes: a statistical study. Org. Geochem..

[41] Harrell JA, Lewan MD (2002). Sources of mummy bitumen in ancient Egypt and Palestine. Archaeometry.

[42] Siddall R (2011). Asphaltite pigments in ancient Egypt. Tradit. Paint News.

[43] Christiansen T (2017). Chemical characterization of black and red inks inscribed on ancient Egyptian papyri: The Tebtunis temple library. J. Archaeol. Sci. Reports.

[44] El Goresy, A. Polychromatic wall painting decorations in monuments of pharaonic egypt: compositions, chronology and painting techniques in The Wall Paintings of Thera. Proceedings of the First International Symposium Thera, Hellas, 30 August-4 September 1997 (ed. Sherratt, S.) 49–70 (Thera Foundation, 2000).

[45] Lee, L. & Quirke, S. *Painting materials in Ancient Egyptian Materials and Technology* (eds. Nicholson, P. T. & Shaw, I.) 104–120 (Cambridge University Press, 2000).

[46] Clark, K. A. *Tracing the evolution of organic balm use in Egyptian mummification via molecular and isotopic signatures*. PhD Thesis, Bristol University (2006).

[47] McCreesh, N. *Ritual Anointing: an investigation into the coatings applied to human hair and coffins in ancient Egypt*. PhD Thesis, University of Manchester (2009).

[48] Serpico, M. *Resins, amber and bitumen in Ancient Egyptian Materials and Technology* (eds. Nicholson, P. T. & Shaw, I.) 430–474 (Cambridge University Press, 2000).

[49] Serpico, M. & White, R. *The use and identification of varnish on New Kingdom funerary equipment in Colour and painting in ancient Egypt* (ed. Davies, W. V.) 33–38 (British Museum Press, 2001).

[50] Colombini MP, Modugno F, Silvano F, Onor M (2000). Characterization of the balm of an Egyptian mummy from the seventh century B.C. Stud. Conserv..

[51] Brettell R, Martin W, Atherton-Woolham S, Stern B, McKnight L (2017). Organic residue analysis of Egyptian votive mummies and their research potential. Stud. Conserv..

[52] Buckley SA, Evershed RP (2001). Organic chemistry of embalming agents in Pharaonic and Graeco-Roman mummies. Nature.

[53] Tchapla A, Méjanelle P, Bleton J, Goursaud S (2004). Characterisation of embalming materials of a mummy of the ptolemaic era. Comparison with balms from mummies of different eras. J. Sep. Sci..

[54] Proefke ML, Rinehart KL (1992). Analysis of an Egyptian mummy resin by mass spectrometry. J. Am. Soc. Mass Spectrom..

[55] Smith, M. *Osiris and the Deceased in UCLA Encyclopedia of Egyptology* (eds. Dieleman, J. & Wendrich, W.). Available at, http://digital2.library.ucla.edu/viewItem.do?ark=21198/zz001nf6bg (2008).

[56] Clark, K. A., Ikram, S. & Evershed, R. P. The significance of petroleum bitumen in ancient Egyptian mummies. *Philos. Trans. R. Soc. A* **374**, 10.1098/rsta.2016.0229 (2016). 10.1098/rsta.2016.0229 PMC5031647 27644983

[57] Maurer J, Möhring T, Rullkötter J (2002). Plant lipids and fossil hydrocarbons in embalming material of Roman Period mummies from the Dakhleh Oasis, Western Desert, Egypt. J. Archaeol. Sci..

[58] Nissenbaum A (1992). Molecular archaeology: organic geochemistry of Egyptian mummies. J. Archaeol. Sci..

[59] Nissenbaum A, Buckley S (2013). Dead sea asphalt in ancient egyptian mummies-why?. Archaeometry.

[60] Rullkötter J, Nissenbaum A (1988). Dead sea asphalt in Egyptian mummies: molecular evidence. Naturwissenschaften.

[61] Spataro M, Millet M, Spencer N (2015). The New Kingdom settlement of Amara West (Nubia, Sudan): mineralogical and chemical investigation of the ceramics. Archaeol. Anthropol. Sci..

[62] Spencer N (2014). Creating and re-shaping Egypt in Kush: responses at Amara West. J. Anc. Egypt. Interconnections.

[63] Spencer, N. & Millet, M. Amara Ouest: aspects de la vie quotidienne au Nouvel Empire en Nubie (Amara West: aspects of daily life in the New Empire in Nubia). *Comptes rendus l’Academie des Inscriptions B.-lett*. **157**, 639–659 https://www.persee.fr/doc/crai_0065-0536_2013_num_157_2_95225 (2013).

[64] Daniels V, Stacey R, Middleton A (2004). The blackening of paint containing Egyptian blue. Stud. Conserv..

[65] Le Fur, D. *La conservation des peintures murales des temples de Karnak* (The conservation of the murals of the temples of Karnak). (Editions Recherche sur les Civilisations (1994).

[66] McCarthy, B. *Technical analysis of reds and yellows in the tomb of Suemniwet, Theban Tomb 92 in Colour and Painting in Ancient**Egypt* (ed. Davies, W. V.) 17–21 (British Museum Press, 2001).

[67] Stacey, R. *Paint media and varnishes in The Nebamun Wall Paintings: Conservation, Scientific Analysis and Display at the British Museum* (eds. Middleton, A. & Uprichard, K.) 51–60 (Archetype in association with the British Museum, 2008).

[68] Stulik, D., Porta, E. & Palet, A. *Analyses of pigments, binding media and varnishes in Art and eternity: the Nefertari Wall Paintings Conservation Project 1986–1992* (eds. Corzo, M. A. & Afshar, M.) 55–65 (Getty Conservation Institute, 1993).

[69] Ménager M, Azémard C, Vieillescazes C (2014). Study of Egyptian mummification balms by FT-IR spectroscopy and GC-MS. Microchem. J..

[70] Łucejko JJ, Lluveras-Tenorio A, Modugno F, Ribechini E, Colombini MP (2012). An analytical approach based on X-ray diffraction, Fourier transform infrared spectroscopy and gas chromatography/mass spectrometry to characterize Egyptian embalming materials. Microchem. J..

[71] Connan, J. La momification dans L’Egypte ancienne: le bitume et les autres ingrédients organiques des baumes de momies ou les ingrédients organiques des baumes de momies égyptiennes; bitume, cires d’abeilles, résines, poix, graisses, huile, vin, etc. (Mummification in Ancient Egypt: bitumen and other organic ingredients from mummy balms or organic ingredients from Egyptian mummy balms; bitumen, beeswax, resins, pitch, grease, oil, wine etc.) in Encyclopédie religieuse de L’Univers végétal. Croyances phytoreligieuses de L’Egypte ancienne (ERUV) III, OrMounsp XVI (ed. Aufrère, S. H.) 163–211 (Université Paul Valéry, 2005).

