# Supplementary material for: Bitumen from the Dead Sea in Early Iron Age Nubia
Source: Sci Rep. 2020 May 20;10:8309. doi: 10.1038/s41598-020-64209-8 (PMC7239913; doi:10.1038/s41598-020-64209-8)
Supplement: Supplementary file 1 — Supplementary Information. [file 41598_2020_64209_MOESM1_ESM.pdf]

Bitumen from the Dead Sea in Early Iron Age Nubia

Kate Fulcher, Rebecca Stacey and Neal Spencer

Supplementary Information

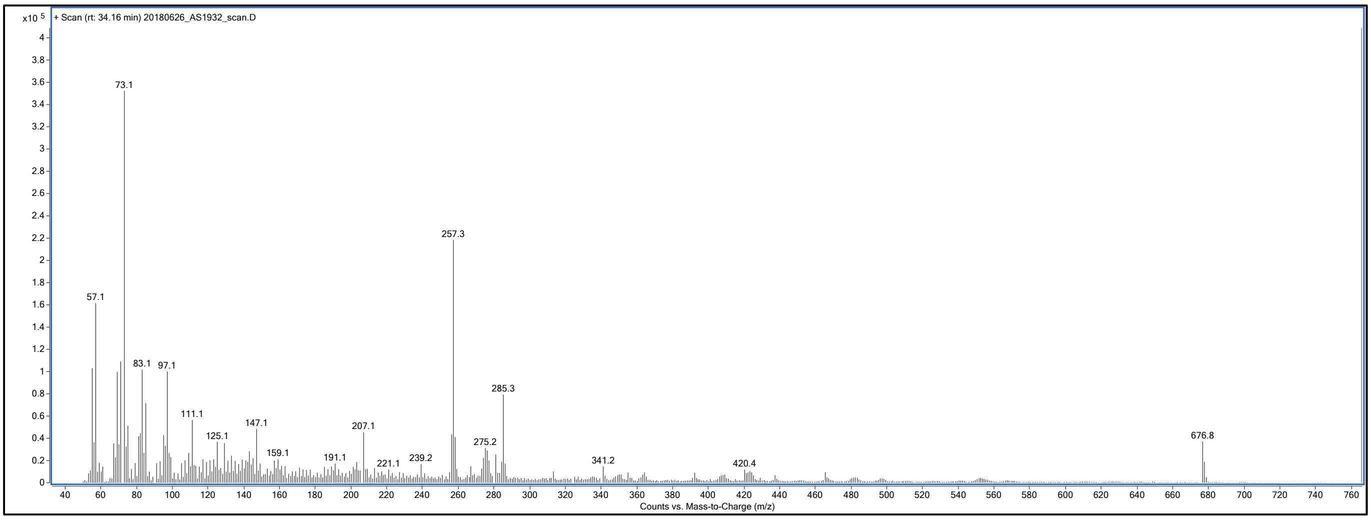

Figure S1. Electron ionization mass spectrum of peak at 34.16 minutes for sample AS1932. Wax with carbon chain length 46 (TMS derivative).

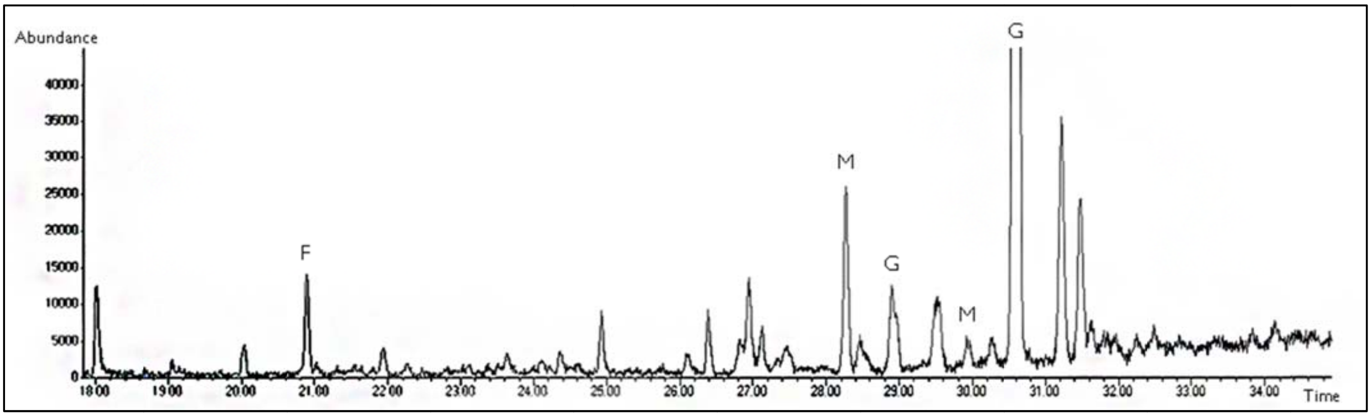

Figure S2. Total ion chromatogram (across 18-35 minutes) showing sugar and uronic acid constituents detected in black paint from palette PS121. F=fucose; X=xylose; M=mannose; G=galactose.

Table S1. Lipidic compounds identified in samples from Amara West using Method B.

Compound identified (TMS derivatives)	AS1932	AS1933	AS1941	AS1948	AS1949	AS1994	PS295
Camphor	y						
Octanoic acid	y	y		y			
Nonanoic acid	y	y	y	y		y	
5-Ethyl-2-methyl-4-furan-3(2H)-one	y	y					
Decanoic acid	y	y		y		y	
Cuparene	y						
Undecanoic acid	y	y					
Dodecanoic acid	y	y		y		y	
Tridecanoic acid	y	y				y	
Tetradecanoic acid	y	y				y	
Pentadecanoic acid	y	y				y	
Hexadecanoic acid	y	y		y		y	y
Heptadecanoic acid	y	y				y	
Octadecanoic acid	y	y		y		y	y
Nonadecanoic acid	y	y		y		y	
Eicosanoic acid	y	y		y		y	
10-Oxooctadecanoic acid	y						
Heneicosanoic acid	y	y		y		y	
Monopalmitin	y	y	y	y	y	y	
Docosanoic acid	y	y		y		y	
Tricosanoic acid	y	y		y		y	
Glycerol monostearate	y	y	y	y	y	y	
Tetracosanoic acid	y	y		y		y	
Hexacosanoic acid	y	y		y		y	
Oleanolic acid	y	y				y	
Moronic acid	y	y				y	
Oleanonic acid	y	y				y	y
Isomasticadienonic acid		y					
Wax ester 42 carbons		y					
Wax ester 44 carbons	y	y					
Wax ester 46 carbons	y	y					
Wax ester 48 carbons	y	y					
Wax ester 50 carbons	y	y				y	

Table S2. Interpretation of biomarker values for Group A samples (friable solid from G321 and palettes PS119, PS152 and PS415).

Biomarker value for Group A	Interpretation
Low Pr/Ph (<1)	Anoxic hypersaline depositional environment, marine carbonate[1,2]
Ts/Tm < 0.5	Marine carbonate[3]
High gammacerane	Hypersaline origin[1,2]
Oleanane low or absent	Marine[2,4]
$C_{31}R/C_{30} > 0.25$	Marine[2]
High C_{35}/C_{34} (>0.8) plus C_{29}/C_{30}>0.6	Marine carbonate[2]
Low diasteranes	Anoxic, marine carbonate[2,5]
Presence of C_{30} steranes	Marine[6]
C_{27}>C_{29} steranes	Marine carbonate[2]
C_{26}/C_{25} tricyclic terpanes < 0.9	Marine[3]

References

1. Mello, M. R., Gaglianone, P. C., Brassell, S. C. & Maxwell, J. R. Geochemical and biological marker assessment of depositional environments using Brazilian offshore oils. *Mar. Pet. Geol*. **5**, 205–223 (1988). https://doi.org/10.1016/0264-8172(88)90002-5

2. Peters, K. E., Walters, C. C. & Moldowan, J. M. The Biomarker Guide (Cambridge University Press, 2005).

3. El Diasty, W. S., Abo Ghonaim, A. A., Mostafa, A. R., El Beialy, S. Y. & Edwards, K. J. Biomarker characteristics of the Turonian-Eocene succession, Belayim oilfields, central Gulf of Suez, Egypt. *J. Assoc. Arab Univ. Basic Appl. Sci*. **19**, 91–100 (2016). https://doi.org/10.1016/j.jaubas.2014.06.001

4. Moldowan, J. M. et al. The molecular fossil record of oleanane and its relation to angiosperms. *Science* **265**, 768–771 (1994). 10.1126/science.265.5173.768

5. Rullkötter, J. & Nissenbaum, A. Dead sea asphalt in Egyptian mummies: molecular evidence. *Naturwissenschaften* **75**, 618–621 (1988). https://doi.org/10.1007/BF00366476

6. Moldowan, J. M., Seifert, W. K. & Gallegos, E. J. Relationship between petroleum composition and depositional environment of petroleum source rocks. *Am. Assoc. Pet. Geol. Bull*. **69**, 1255–1268 (1985). https://doi.org/10.1306/AD462BC8-16F7-11D7-8645000102C1865D
